# Sexual health needs of people living with HIV in a rural area of central India

**DOI:** 10.4103/2589-0557.75021

**Published:** 2010

**Authors:** Quazi Syed Zahiruddin, A. M. Gaidhane, Lalit Waghmare, Nazli Khatib, Shilpa Gaidhane, Tripti Srivatsava, S. P. Zodpey

**Affiliations:** Department of Community Medicine, Datta Meghe Institute Medical Sciences, Nagpur, Maharashtra, India; 1 Department of Physiology, Datta Meghe Institute Medical Sciences, Nagpur, Maharashtra, India; 2 Government Medical College, Nagpur, Maharashtra, India; 3 Public Health Education, Public Health Foundation India, New Delhi, India

Sir,

Policymakers, donors, researchers, and activists should recognize the benefits of supporting the fuller integration of HIV prevention efforts with reproductive health services.[1,2] Not enough data are available on the reproductive and sexual health of HIV-infected people of the reproductive age group in India,[3] in spite of being having the second largest number of people living with HIV in the world.[4] This cross-sectional study was conducted in a tertiary care teaching hospital in a rural area of central India with an objective to identify the sexual health needs and practices of people of the HIV-positive group and determinants for access to HIV treatments, and safer sex practices.

Considering the operational difficulties, like confidentiality and social stigma, random sampling was difficult. Therefore, all people who were HIV seropositive and attending the hospital, over a period of 6 months, were approached. The average number of years since the diagnosis of HIV status was 3.1 years. Males contacted more health care providers as compared to females. With regard to the sexual encounter, 43 (73 %) said that they indulged in sex in the last 1 month. A total of 12 of the 16 female participants said that they had sexual encounter in the last 1 month with their husband; however, the usual frequency was at least once a week.

Out of 31 male participants, around 16% had more they had sex more than once and almost 10 male participants, in spite of being HIV positive, indulged in the high-risk activities, i.e., they were having nonsteady/nonregular partners. The study reveals a shocking fact that in spite of being HIV seropositive for an average of 3.1 years, and having an average 3.9 times contact with health care services, almost 76.2% of the participants were never told about the correct and consistent condom use by the health care workers/counselors. Only 20 (17.82%) correctly demonstrated condom use on a penis model by the health care workers/counselors.

Less than half of the participants (45.7%) participants said that they used the condom during the last sexual act. Only 5 (31.2%) women had mentioned that their partner used condom the last time they had sex; this could be because of lack of condom negotiation skills among the women, and females are perceived as passive partners by their husbands.

Reasons for an infrequent condom use were as follows: males feeling uncomfortable using it (83%), condoms being not available when needed (42%), and the reduced sexual pleasure on using condoms (64%). However, a significant proportion (71%) of respondents mentioned, “We both are HIV positive so in no way condom will be helpful to us.” Services should be more integrated, and referrals and linkages can be strengthened. Finding in this study reveals that sexual health needs require more attention while planning.[5]
